# Emotion Regulation Strategies and Sense of Life Meaning: The Chain-Mediating Role of Gratitude and Subjective Wellbeing

**DOI:** 10.3389/fpsyg.2022.810591

**Published:** 2022-05-17

**Authors:** Zhengzheng Lin

**Affiliations:** School of Educational Studies, Universiti Sains Malaysia, Penang, Malaysia

**Keywords:** emotion regulation strategies, gratitude, subjective wellbeing, sense of life meaning, college students

## Abstract

This study aimed to explore the mechanism of college students’ meaning of life. The Emotion Regulation Questionnaire, the Gratitude Questionnaire Six-Item Form, the General Wellbeing Schedule, the Meaning in Life Questionnaire were used as measurement instruments. In total, 1,312 valid responses were obtained. The results showed that the cognitive reappraisal and expression suppression strategies were significantly positively and negatively correlated with gratitude, subjective wellbeing, and the sense of life meaning, respectively. Further, Emotion regulation strategies can affect college students’ sense of life meaning through three paths: the mediating effect of gratitude; the mediating effect of subjective wellbeing; the chain mediating effect of gratitude and subjective wellbeing. This study illuminated the roles of gratitude, and subjective wellbeing in influencing the sense of life meaning among the Chinese college students. Limitations and future research directions are discussed.

## Introduction

College students encounter important life issues such as professional education, adaptation, making friends, and choosing careers. However, due to undeveloped mind, they are prone to psychological conflicts, leading to great mental pressure and loss of confidence in life. Many students incompletely experience the meaning of life, even some of them experience existential crisis and extreme suicidal ideation. Psychological problems and suicidal behaviors of college students originate from the lack of sense of life meaning, which is an indicator of mental health (Kleiman et al., 2013a). Therefore, it is very important to explore the source of sense of life meaning, and to explore ways and measures to enhance it.

Emotion is the core driving force of life that can sustain growth (Campos et al., 1989). A good mood can promote the development of physical and mental health. Emotional dysfunction occurs when an individual’s emotions are uncoordinated with their life situation. The Chinese National Mental Health Development Report (2019–2020) noted that 8.4 and 18.5% of college students have a tendency to suffer from depression and anxiety, respectively; moreover, 4.2% are at high risk of depression (Fu and Zhang, 2021). Positive emotions help increase perception of meaning in life (Fredrickson et al., 2000; Hicks et al., 2012). Students need to adjust the their emotions to avoid experiencing the negative ones in order to obtain enhanced life adaptation. Emotion regulation is the internal key mechanism of individual development. Individuals use different strategies to control the emotions that affect their occurrence. It is the process of monitoring, evaluating, and regulating the occurrence, experience, and expression of emotions so that individuals can better adapt for surviving and achieving their goals in emotionally arousing situations (Thompson, 1994; Meng, 2005).

Gratitude, an important concept in the field of positive psychology, was referred to as the psychological tendency of individuals to recognize that they have received valuable favors or help from the outside world and are willing to reciprocate. Current research generally regards gratitude as an emotional trait (Rosenberg, 1998; McCullough et al., 2002). Generation of gratitude depends on the individual being able to recognize the value and meaning of the object of gratitude (Adler and Fagley, 2005; Disabato et al., 2017). Individuals with high gratitude tendencies have a higher perception of the purpose and meaning of life (Wood et al., 2008; Lin, 2021).

Wellbeing is a subjective experience; it is the overall evaluation and feeling toward the quality of life based on the standards set by oneself, consisting of two parts: cognitive component and emotional component. Cognitive component refers to life satisfaction (Diener, 1984). Individuals with high wellbeing can balance their positive and negative emotions well, and have more energy to explore the world, explore themselves, and seek the meaning of life, so as to gain more experience of the meaning of life (Ryff and Singer, 2008; Yin et al., 2019).

Thus far, college students’ sense of life meaning and the internal mechanism of the emotion regulation strategies affecting it have been insufficiently discussed. Therefore, this study examined the influence of emotion regulation strategies on college students’ sense of life meaning. Further, it assessed the independent and chain mediation effects of gratitude and subjective wellbeing on the impact of emotion regulation strategies on the sense of meaning of life, with an aim to reveal the effect of the former on the latter. Simultaneously, it provided effective suggestions for college students to rationally use emotion regulation strategies for improving their sense of happiness and alleviate the impact of negative emotions on the sense of life meaning.

## Literature Review

### Emotion Regulation Strategies and Sense of Life Meaning

Masters (1991) believed that emotion regulation strategies are the methods used by people to regulate their emotions in a conscious and planned manner. Gross (1999, 2001) proposed that affects the kind of emotion an individual feels, when it occurs, and how to experience and express it.

Emotion regulation process theory pointed out that there are two most commonly used and valuable emotion regulation strategies in daily life: cognitive reappraisal and expressive suppression. The two have different effects on the adjustment of emotion, cognition and social behavior. Expression suppression is associated with negative outcomes and cognitive reappraisal is associated with positive outcomes (Gross, 1998a,b, 1999, 2001).

The cognitive reappraisal strategy occurs in the early stage of emotions. Through re-understanding and evaluation of emotional events, the reaction produced by them can be alleviated. It can effectively reduce negative emotions and physical stress (Sheppes et al., 2011) as well as help individuals make effective decisions (Heilman et al., 2010). Moreover, it changes the perception of personal meaning of such occurrences to positively impact social behaviors. As an effective technique to suppress negative emotions, it helps to improve life satisfaction (Gong et al., 2013); moreover, it is closely related to wellbeing and mental health (Boden et al., 2012; Xu et al., 2020).

The expression suppression strategy entails suppressing and avoiding the emotion’s expression after the individual experiences it. It does not reduce the psychological experience produced by a negative emotion, and is a non-adaptive method. Individuals who are accustomed to using expression suppression strategies have relatively more and fewer negative and positive experiences, respectively (Wang and Guo, 2003), thereby reducing the level of mental health. Expression suppression requires the consumption of cognitive resources and negatively impacts other cognitive activities, emotional experiences, and behaviors (Julian and Richard, 2000; Gross, 2002; Ochsner et al., 2002; Garnefski et al., 2004).

Studies have demonstrated that cognitive reappraisal strategies are superior to expression suppression in maintaining physical and mental health (Gross and John, 2003; Moore et al., 2008; Hughes et al., 2011). The former can reduce symptoms of depression to some extent and help individuals better cope with life (Garnefski and Kraaij, 2011).

The first psychologist to conduct systematic research on the sense of life meaning was Viktor Frankl, who believed that everyone needs the meaning of existence, has the motivation to constantly search for it, and persistently explores it and the value of life. If people stop exploring the sense of life’s meaning, it will produce spiritual emptiness and cause psychological problems. The sense of life meaning refers an inner psychological experience, entailing people experiencing and comprehending the meaning of their lives, while also recognizing their the goals and life missions (Crumbaugh, 1973; Steger et al., 2009).

Tamirb (2016) revealed that cognitive reappraisal is highly correlated with sense of life meaning. A model constructed by Zhu et al. (2017) demonstrated that cognitive reappraisal strategy plays a positive role in sense of life meaning. Therefore, combing the theoretical perspectives, Hypothesis 1 was derived as follows:

H1: Emotion regulation strategies significantly predict the sense of life meaning.

### Emotion Regulation Strategies, Gratitude, and Sense of Life Meaning

Gratitude as an emotional trait, according emotion regulation process theory, different specific strategies of emotion regulation will have different effects on emotions. Gratitude mediated the link of emotion regulation to burnout.

Gratitude as a positive resource buffer the effects of cognitive change on emotional exhaustion (Guan and Jepsen, 2020).

The internal and external goal theory of gratitude describes it as closely related to self-management. Individuals with a high level of gratitude pay greater attention to a task’s meaning and value. They exert more effort to achieve internal goals, while being less directed toward materialistic objectives. Furthermore, they can experience happiness and lead individuals to perceive a deep sense of meaning (Bono and Froh, 2009). Fredrickson (2001) employed his broaden-and-build theory to study gratitude. Gratitude can expand and construct individual cognitive levels and opportunities to build resources, magnify the beautiful things in life, and actively feel the sense of life meaning; thus, gratitude promotes enhanced development and adaptation of persons. Moreover, it can effectively buffer the adverse effects of external pressures on the individual, form an adaptive response to negative events, expand the individual’s timely thinking and behavior paradigm to efficiently trigger positive reactions, seek self-worth, and gain more happiness (Wood et al., 2008; Li, 2016). Gratitude, as a protective factor, plays a regulatory role in enhancing the sense of life meaning and reducing the risk of suicide; additionally, it can be utilized as a valuable intervention to enrich the sense of life meaning (Kleiman et al., 2013b; Tongeren et al., 2015).

The mediating mechanism of gratitude in emotion regulation strategies and the sense of life is unclear. However, strong evidence has demonstrated the relationship between it and the latter. Therefore, gratitude is expected to play a mediating role between the aforementioned two variables. Hypothesis 2 was formulated as follows:

H2: Gratitude plays a mediating role between emotion regulation strategies and the sense of life meaning.

### Emotion Regulation Strategies, Subjective Wellbeing, and Sense of Life Meaning

Subjective wellbeing plays a key role in human health and social adaptation (Liu et al., 2013). Empirical research has reported that the cognitive reappraisal and expression suppression strategies are related to high and low happiness, respectively (Haga et al., 2009; Balzarotti et al., 2016). Cognitive reappraisal strategies have a positive effect on human advanced emotions, such as subjective wellbeing, life satisfaction (Gross and John, 2003). Individual who use cognitive reappraisal strategies more often feel more satisfaction, more positive emotions, and less negative emotions in their lives, and they are more able to maintain a positive attitude in the face of stressful situations, and they are able to re-understand and recognize stressful events, positive efforts to change negative emotions. Expression suppression strategies leads to lower subjective wellbeing and increased negative emotional experience (Dryman and Heimberg, 2018).

Researchers have conducted empirical studies on the relationship between subjective wellbeing and sense of life meaning, but have not achieved consistent results (Shrira et al., 2011; Li et al., 2021). Shrira et al. (2011) showed that subjective wellbeing and meaning in life are likely to compensate for each other. A meta-analysis based on a Chinese sample showed the sense of life meaning to be significantly positively correlated with subjective wellbeing, life satisfaction, and positive emotions (Jin et al., 2016). Li et al. (2014) revealed that College students’ wellbeing index positively predict the sense of life meaning. Wellbeing can promote individual coping with meaning and perception of meaning in life. From the literature above, Hypothesis 3 was formulated as follows:

H3: Subjective wellbeing plays a mediating role between emotion regulation strategies and the sense of life meaning.

### Emotion Regulation Strategies, Gratitude Subjective Wellbeing, and Sense of Life Meaning

Gratitude is a positive emotional trait that helps people construct lasting personal resources, promotes individual happiness and personal growth, changes cognition, and increases individual pursuit and possession of the sense of life meaning. Life satisfaction is a main indicator for measuring subjective wellbeing. Several studies have demonstrated that gratitude, as a positive variable, is closely related to life satisfaction (Lyubomirsky et al., 2005; Wood et al., 2007). It is significantly and positively correlated with subjective wellbeing, as confirmed by previous empirical research (Watkins et al., 2003; Chan, 2013; Witvliet et al., 2018). McCullough et al. (2002) conducted a study with college students and found that individuals with a higher tendency to gratitude have greater life satisfaction and a more optimistic and energetic attitude toward life.

Considering the in-depth study of subjective wellbeing, researchers are not restricted to the direct impact of emotion regulation strategies on it; they can explore the internal mechanisms of the two effects and possible intermediary factors. Studies have found that emotion regulation strategies can affect subjective wellbeing through internal factors such as individual mental flexibility and self-esteem (Liu et al., 2015; Chai et al., 2018). Therefore, how to mobilize positive internal resources such as subjective wellbeing through these strategies is an important way to enhance the sense of life meaning. Broaden-and-build theory and related research suggests that there may be some mediation between gratitude and a sense of meaning in life. Gratitude manifests its extended construction effect by acting on these mediating variables, thereby affecting the individual’s sense of life meaning (Fredrickson, 2001).

Furthermore, Watkins et al. (2003) reached a consistent conclusion and pointed out that there is a mutually reinforcing effect between gratitude and wellbeing. In summary, emotion regulation strategies, such as the cognitive reappraisal ones, play a valuable role in promoting human high-level emotions, such as subjective wellbeing. Simultaneously, gratitude and subjective wellbeing, as positive emotions, can help the individual’s coping style and the perception of the life meaning (King and Hicks, 2006; Chu et al., 2019).

If there are multiple mediations in the mediation model that are interrelated, the chain mediation can occur (Hayes, 2013). Therefore, we systematically explored the intermediary relationship between gratitude and subjective wellbeing in the influence of emotion regulation strategies on the sense of life meaning. Hypothesis 4 was derived as follows:

H4: Emotional regulation strategies have an impact on the sense of life meaning through the chain intermediary role of gratitude and subjective wellbeing.

### Hypothetical Model

**FIGURE 1:**
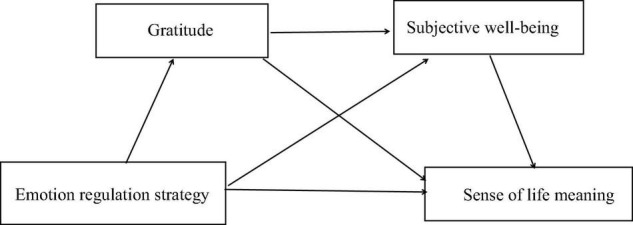
Hypothetical relationship model.

According to the aforementioned theories and studies, Figure 1 provides a diagram of the hypothetical model. In the mode, Emotion regulation strategies is assumed to predict the sense of life meaning of college students, and gratitude and subjective wellbeing are the two chain-mediating factors in this relationship.

## Materials and Methods

### Participants

Using the cluster sampling method, freshmen to senior students from an undergraduate college in the Fujian Province of China were selected as the survey participants. College counselors help investigator recruit participants. Respondents were told that their data would remain confidential. The questionnaires were filled out after obtaining informed consent from the participants.

Overall, 1,400 questionnaires were distributed and recovered; 88 questionnaires were eliminated due to missing answers, while 1,312 valid questionnaires were obtained, with an effective response rate of 93.71%. There were 588 males and 724 females; 435, 389, 310, and 178 were freshmen, sophomores, juniors, and seniors, respectively. Furthermore, 246 were only-child and 1,066 had siblings; 1,130 and 182 were from rural and urban areas, respectively. The average age of the participants was 19.26 years (*SD* = 1.15). The age distribution ranged from 17 to 24 years (Table 1).

**TABLE 1:** Demographic distribution of the sample.

	Groups	N	%	*M* (*SD*)
Age	17–25 years old			19.26 (1.15)
Sex	Male	588	44.8	
	Female	724	55.2	
Academic year	First year	435	33.2	
	Second year	389	29.6	
	Third year	310	23.6	
	Forth year	178	13.6	
Only-child	YES	246	18.8	
	No	1,066	81.3	
Birthplace	Rural	1,130	86.1	
	Urban	182	13.9	

### Measures

#### Emotional Regulation Strategy Scale

The Emotion Regulation Questionnaire (ERQ) was compiled by Gross and John (2003), and revised by Wang et al. for use with a Chinese sample (Wang et al., 2017). It has 10 items that are divided into 2 dimensions: cognitive reappraisal and expression suppression. Each dimension includes the regulation of positive emotions and negative emotions. The items are rated on a seven-point Likert scale, ranging from 1 “*completely disagree*” to 7 “*completely agree*.” In this study, the Cronbach’s α was 0.753.

#### Gratitude Scale

The six-item Chinese version of the Gratitude Questionnaire Six-Item Form(GQ-6) was compiled by McCullough et al. (2002) and revised by Wei et al. (2011). It has five levels of scoring; the higher the score, the greater the tendency to be grateful. In this study, the Cronbach’s α was 0.622.

#### Subjective Wellbeing Scale

The General Wellbeing Schedule (Fazio, 1977) was developed by the National Center for Health Statistics in 1977 to evaluate happiness; moreover, Duan revised its Chinese version (Duan, 1996). Many previous studies used this tool to measure an individual’s subjective wellbeing. In this scale, the subjective wellbeing is divided into six dimensions, including concerns about health, energy, satisfaction and interest in life, melancholy or pleasant mood, control of emotions and behaviors, and relaxation and tension. Of the total 18 items, questions 2, 5, 6, and 7 use a 5-point scoring method, while 15–18 employ a 10-point one; the remaining questions utilize a 6-point scoring system. The higher the score, the greater the happiness index. In this research, the Cronbach’s α was 0.791.

#### Sense of Life Meaning Scale

Liu and Gan (2010) revised the Meaning in Life Questionnaire compiled by Steger et al. (2006). It has high reliability and validity and is widely used in the Chinese context. The scale is composed of two subscales: having a sense of meaning and seeking it. The higher the score, the stronger the sense of life meaning. In this study, the Cronbach’s α was 0.842.

#### Analytical Method

The SPSS 21.0 software was employed to perform the descriptive statistics and the correlation analysis for each variable. The PROCESS program developed by Hayes and the non-parametric percentile bootstrap were used to examine the chain mediating role of gratitude and subjective wellbeing in the relationship between the emotion regulation strategies and the sense of life meaning (Hayes, 2013).

## Results

### Common Method Bias Test

Harman’s single factor test was used to assess common method bias. Overall, 10 factors with eigenvalues greater than 1 were found; the variance explained by the first factor was 22.75%, which was less than the critical standard of 40%. Thus, common method bias was excluded from this study.

### Descriptive Statistics and Correlation Analysis

As shown in Table 2, the descriptive statistics indicated that the college students’ gratitude, subjective wellbeing, and the sense of life meaning were at an intermediate level. Additionally, the correlation analysis reported a significant positive correlation between the cognitive reappraisal strategy, gratitude, subjective wellbeing, and the sense of life meaning. There was a significant negative correlation between the expression suppression strategy and gratitude, subjective wellbeing, and the sense of meaning life.

**TABLE 2:** Means, standard deviations, and Pearson correlation.

	*M* ± *SD*	1	2	3	4
Cognitive reappraisal strategy	4.45 ± 0.93	−	0.086**	0.380***	0.294***
Expression suppression strategy	3.60 ± 0.73	0.086**	−	−0.139***	−0.233***
Gratitude	3.77 ± 0.56	0.380***	−0.139***	−	0.396***
Subjective wellbeing	4.45 ± 0.71	0.294***	−0.233***	0.396***	−
Sense of life meaning	5.07 ± 0.94	0.358***	−0.160**	0.474***	0.384***

**p < 0.01, ***p < 0.001.

### Chained Mediating Analyses

Under the condition of controlling gender and grade, the regression analysis results of gratitude and subjective wellbeing in the cognitive reappraisal strategy and the sense of meaning life are shown in Table 3. The cognitive reappraisal strategy had an impact on the sense of life meaning. It had a significant positive predictive effect (β = 0.355, *p* < 0.001), and the sense of meaning of life had a direct positive predictive effect on gratitude (β = 0.366, *p* < 0.001) and subjective wellbeing (β = 0.166, *p* < 0.001). Gratitude had a significant positive predictive effect on subjective wellbeing (β = 0.334, *p* < 0.001); when cognitive reappraisal strategies, gratitude, and subjective wellbeing predicted the sense of life meaning simultaneously, they had a positive predictive effect on the sense of life meaning (β = 0.176, *p* < 0.001; β = 0.332, *p* < 0.001; β = 0.203, *p* < 0.001, respectively).

**TABLE 3:** Regression analysis of the cognitive reappraisal strategy and the sense of life meaning regarding the chain intermediary model.

Dependent variable Independent variable	Sense of life meaning		Gratitude		Subjective wellbeing		Sense of life meaning	
	β	*t*	β	*t*	β	*t*	β	*t*
Gender	0.016	0.620	0.123	4.827***	–0.029	–1.134	–0.027	–1.153
Grade	–0.024	–0.947	–0.030	–1.199	–0.075	−3.019**	0.003	0.127
Cognitive reappraisal strategy	0.355	13.675***	0.366	14.341***	0.166	6.149***	0.176	6.886***
Gratitude					0.334	12.255***	0.332	12.392***
Subjective wellbeing							0.203	7.870***
*R*	0.359		0.401		0.433		0.544	
Δ*R*^2^	0.127		0.159		0.185		0.294	
*F*	64.711***		83.420***		75.293***		109.988***	

**p < 0.01, ***p < 0.001.

Table 4 displays the analysis results of the mediating effects of gratitude and subjective wellbeing on expression inhibition strategies and the sense of meaning of life under the conditions of controlling grade and gender. Expression suppression was found to have a significant negative impact on the sense of life meaning. The predictive effect (β = -0.155, *p* < 0.001) of the sense of life meaning, and had a significant negative predictive effect on gratitude (β = -0.123, *p* < 0.001) and subjective wellbeing (β = -0.180, *p* < 0.001); the latter had a significant positive predictive effect on subjective wellbeing (β = 0.373, *p* < 0.001). When expression suppression strategy, gratitude, and subjective wellbeing predicted the sense of life meaning simultaneously, expression suppression strategies had a significant negative effect on the sense of life’s predictive effect (β = -0.058, *p* < 0.05). Furthermore, gratitude and subjective wellbeing both had positive predictive effects on the sense of life meaning (β = 0.383, *p* < 0.001; β = 0.220, *p* < 0.001, respectively).

**TABLE 4:** Regression analysis of the expression suppression strategy and the sense of life meaning regarding the chain intermediary model.

Dependent variable Independent variable	Sense of life meaning		Gratitude		Subjective wellbeing		Sense of life meaning	
	β	*t*	β	*t*	β	*t*	β	*t*
Gender	0.039	1.436	0.150	5.531***	–0.034	–1.351	–0.023	–0.959
Grade	–0.034	–1.263	–0.043	–1.580	–0.072	2.898**	0.001	0.054
Expression suppression strategy	–0.155	−5.637***	–0.123	−4.508***	–0.180	−7.167***	–0.058	−2.375*
Gratitude					0.373	14.693***	0.383	14.701***
Subjective wellbeing							0.220	8.378***
*R*	0.168		0.209		0.442		0.523	
Δ*R*^2^	0.026		0.041		0.193		0.271	
*F*	12.723***		19.820***		79.347***		98.531***	

*p < 0.05, **p < 0.01, ***p < 0.001.

**FIGURE 2:**
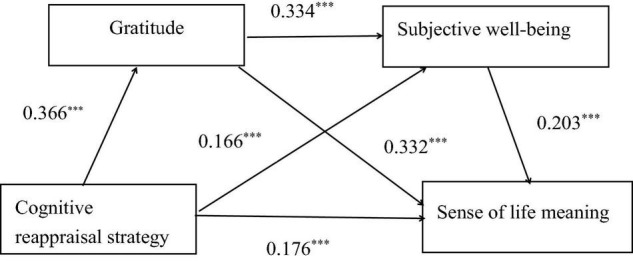
Diagram of the chain mediation. ^***^p < 0.001.

In order to further test the mediating effect of gratitude and subjective wellbeing on cognitive reappraisal strategy and sense of life meaning, the PROCESS macro version 3.3 for SPSS (model 6) was employed for the chain mediation analysis (Hayes, 2015). The bootstrapping method was utilized to repeat the sample 5,000 times to calculate for a 95% confidence interval (CI). The results are displayed in Table 5. The mediating effect of gratitude and subjective wellbeing was significant, with a mediating effect value of 0.1857. Specifically, the impact of the cognitive reappraisal strategy on the sense of life meaning in college was affected by three indirect effects, all of which reached a significant level: First, regarding the indirect effect 1 consisting of the cognitive reappraisal strategy → gratitude → sense of life meaning (0.1255), the 95% confidence interval was [0.0990, 0.1577], excluding 0, indicating that the mediating role of gratitude was significant. Second, for the indirect effect 2 through cognitive reassessment → gratitude →subjective wellbeing → sense of life meaning (0.0259), the 95% confidence interval [0.0176, 0.0361] excluded 0, indicated that gratitude and subjective wellbeing played a significant role in the chain mediation between cognitive reappraisal strategy and sense of life meaning. Third, regarding the indirect effect 3 (0.0343) consisting of the cognitive reappraisal strategy → subjective wellbeing → the sense of life meaning, the 95% confidence interval [0.0202, 0.0516] did not contain 0, indicating that the mediating effect of subjective wellbeing was significant. Figure 2 presents the specific path through which the undergraduates’ cognitive reappraisal strategy affects the sense of life meaning.

**TABLE 5:** The mediating effect of gratitude and subjective wellbeing on cognitive reappraisal strategy and sense of life meaning.

	Effect	Boot SE	BootLLCI	BootULCI	Relative mediation effect (%)
Total indirect effect	0.1857	0.0161	0.1563	0.2192	51.43
1 → 2 → 4	0.1255	0.0150	0.0990	0.1577	34.75
1→ 2 → 3 → 4	0.0259	0.0046	0.0176	0.0361	7.17
1 → 3 → 4	0.0343	0.0078	0.0202	0.0516	9.50

*1, cognitive reappraisal strategy; 2, gratitude; 3, subjective wellbeing; 4, sense of life meaning.*

**FIGURE 3:**
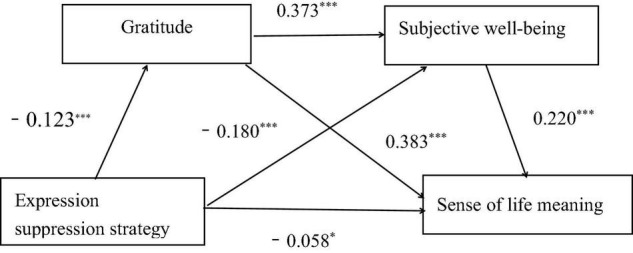
Diagram of the chain mediation. *p < 0.05 and ^***^p < 0.001.

The mediating effect of gratitude and subjective wellbeing on expression suppression strategy and the sense of life meaning is shown in Table 6. The total indirect effect was −0.1334; moreover, the influence of expression suppression strategies on the sense of life was indirectly affected by three paths. First, the indirect effect 1 (−0.0674) consisting of expression suppression strategy → gratitude → the sense of life meaning, with a 95% confidence interval of [−0.0982, −0.0406] excluding 0, indicated that the mediating effect of gratitude was significant. Second, for the mediating effect 2 (−0.0146) comprising expression suppression strategy → gratitude → subjective wellbeing → the sense of life meaning, the 95% confidence interval [−0.0230, −0.0083] excluded 0, indicating gratitude and subjective wellbeing play a significant chain intermediary role between expression suppression strategies and the sense of life meaning. The chain mediation effect between expression suppression strategy and the sense of life was significant. Third, regarding the indirect effect 3, composed of expression suppression strategy→ subjective wellbeing → the sense of life meaning 3 (−0.0514), the 95% confidence interval [−0.0750, −0.0335] did not contain 0, indicating that the mediating effect of subjective wellbeing was significant. The specific path through which the undergraduates’ expression suppression strategy affects the sense of meaning in life is shown in Figure 3.

**TABLE 6:** The mediating effect of gratitude and subjective wellbeing on the expression suppression strategy and the sense of life meaning.

	Effect	Boot SE	BootLLCI	BootULCI	Relative mediation effect (%)
Total indirect effect	–0.1334	0.0197	–0.1749	–0.0976	65
1→ 2 → 4	–0.0674	0.0146	–0.0982	–0.0406	32.85
1→ 2 → 3 → 4	–0.0146	0.0037	–0.0230	–0.0083	7.12
1 → 3→ 4	–0.0514	0.0105	–0.0750	–0.0335	25.05

*1, expression suppression strategy; 2, gratitude; 3, subjective wellbeing; 4, sense of life meaning.*

## Discussion

### Descriptive Statistics and Correlations

This study divided emotion regulation strategies into cognitive reappraisal and expression suppression. The results indicated that the former was significantly positively correlated with the sense of life meaning in college students; moreover, it significantly positively predicted the sense of life meaning. This was consistent with the results of previous studies (Zhu et al., 2017). However, expression suppression strategy was significantly negatively correlated with the sense of meaning of life; additionally, it significantly negatively predicted the sense of life meaning. Simultaneously, it demonstrated that the two emotion regulation strategies had different mechanisms and effects that may be related to the consumption of cognitive resources (Wang and Guo, 2003). Cognitive reappraisal occurs when individuals readjust their cognition before the emotions occur, thus changing their understanding of the emotional events and reducing the psychological experience of negative emotions. It consumes less cognitive resources and can acquire a positive emotion regulation ability; therefore, it can actively experience the meaning of existence. Expression inhibition occurs when emotions are awakened, consciously hindering one’s own emotional expression behavior. It needs to participate in the whole process of emotion occurrence, in which it greatly consumes cognitive resources and affects the positive emotional experience’s influence. Simultaneously, it easily produces meaninglessness (Peng et al., 2011). Additionally, it confirms the conclusions of previous studies. Cognitive reappraisal can significantly change a person’s emotional experience, however, the effect of expression suppression is relatively poor, with lower positive and negative emotional experiences (Chen et al., 2009).

As a protective factor, the cognitive reappraisal strategy is an effective technique for emotion regulation and is superior to the expression suppression strategy (Hughes et al., 2011).

### The Mediating Role of Gratitude

The results demonstrated that for both the cognitive reappraisal and expression suppression strategies, gratitude plays a mediating role between the emotion regulation strategies and the sense of meaning in life. Thus, it can be considered that gratitude is important for the sense of meaning in life. The cognitive reappraisal strategy can be employed as an effective way for regulating emotions, helping individuals disaffiliate from negative frustration incidents, evaluate events from a rational and objective perspective, and enhance positive emotional experiences such as gratitude. According to the extended construction theory of gratitude, gratitude can improve individual cognition as well as help re-recognize and interpret meaning. Reminding people about gratitude occasionally can resist the impact of negative emotions on mental health (Kumar and Epley, 2018), thereby enhancing the perception and experience of the value of one’s own existence. However, if an individual uses the expression suppression strategy for a prolonged period, they report a lower level of gratitude. Due to the habitual suppression of one’s negative emotions, it consumes more cognitive resources, which is not conducive to the generation of positive emotions and physical and mental health.

### The Mediating Role of Subjective Wellbeing

Studies have confirmed the mediating role of subjective wellbeing on the relationship between emotion regulation strategies and the sense of meaning in life. The two emotion regulation strategies of cognitive reappraisal and expression suppression have different effects on the latter by regulating subjective wellbeing. This is consistent with the results of previous research (Srivastava et al., 2009; Cutuli, 2014; Kobylińska et al., 2020).

The cognitive reappraisal strategy promotes the level of the sense of life meaning through subjective wellbeing, while the effect of expression suppression is contrary. Those students who tend to re-evaluate cognitively have greater positive emotional experiences and behaviors. Individuals internally use cognitive reappraisal strategies to construct positive perceptions of life events, thereby promoting happiness. Accordingly, they have sufficient energy to explore the world and discover themselves, which to a certain extent enhances the college students’ understanding and experience of the sense of life meaning (Quoidbach et al., 2015; Szczygie and Mikolajczak, 2017). Gross and John (2003) investigated the relationship between the two emotion regulation strategies and wellbeing and depression. Cognitive reappraisal and expression suppression were related to positive and negative outcomes, respectively. It can be observed that different emotions have varying regulating effects that will lead to discrepancies in the impact of subjective wellbeing on the sense of meaning in life.

### Chain-Mediating Effect of Gratitude and Subjective Wellbeing

The research results indicated that gratitude and subjective wellbeing play a chain-like mediating role between the emotion regulation strategies and the sense of meaning in life. College students experience major changes in their status, roles, and living environment; moreover, higher requirements are placed on their adaptability and self-regulation. If the emotion regulation strategies cannot be employed rationally, strong psychological conflicts are likely to occur, thereby reducing the experience of the sense of life meaning. Fredrickson believed that positive emotional states can broaden the categories of attention and cognition as well as stimulate individuals to explore the meaning of existence (Strumpfer, 2006). Additionally, gratitude and subjective wellbeing, as positive emotions, have also been found to positively impact an individual’s physical and mental health or behavioral response. For example, people with high gratitude experience less loneliness and depression (Fan and Wu, 2020). Simultaneously, individuals can give meaning to life through subjective wellbeing (Xie and Zou, 2013). In previous studies on the relationship between emotions and other variables, the focus was on the former’s internal mechanism as a whole, and the uniqueness of the different emotion regulation strategies in the process was disregarded. This study’s findings demonstrated that emotion regulation strategies can influence college students’ sense of meaning in life through the chain mediation of gratitude and subjective wellbeing. This indicated that the use of the emotion regulation strategies can affect their gratitude and subjective wellbeing, and subsequently impact the sense of life meaning. This result reflected the close connection between the four variables. However, it is worth mentioning that the cognitive reappraisal and expression suppression strategies had different effects on the sense of meaning of life through gratitude and subjective wellbeing. Therefore, the relationship model formed was distinct as well.

The cognitive reappraisal strategy can adjust one’s emotions by changing the cognitive evaluation of events. When encountering a negative emotional incident, by using a positive perspective to give the event a new meaning, this emotional processing method will transfer to the experience of life. The expansion construction theory of gratitude proposes that gratitude can help individuals improve their cognitive styles, absorb more positive signals from life, broaden effective and lasting psychological resources, obtain greater happiness, and deepen the understanding and experience of the sense of life meaning. The expression suppression strategy prevents one’s emotional expression behavior through self-control. Further, emotional suppression conceals the expressive response. Although it can bring a certain effect in the short term, it does not reduce the negative emotional experience; moreover, the motivation of negative emotions is not weakened (Gross, 1998b; Brenning et al., 2015). According to the “water pressure model” of emotions, inappropriate or long-term inhibition of negative emotional expression will lead to an increase in the intensity of negative subjective experiences (Huang and Guo, 2002). Therefore, if strong negative emotions are only suppressed and are not effectively weakened, rebounds will occur easily, leading to a stronger negative psychological experience, which in turn affects the perception of the sense of life meaning.

## Conclusion

This study outcomes show that cognitive reappraisal strategy serves as a significant positive predictor of the sense of life meaning. While expression suppression strategy serves as a significant negative predictor. Emotion regulation strategies can affect college students’ sense of life meaning through the chain mediating effect of gratitude and subjective wellbeing.

## Implications and Suggestions

This study offers the following implications:

First, the cognitive reappraisal strategy is a positive factor that promotes the growth of college students. However, the long-term use of the expression suppression strategies is more likely to produce emotion regulation disorders (Chen et al., 2009); therefore, they should be used with caution. Regarding the role of cognitive reappraisal, it is necessary to pay attention to and effectively guide students’ emotional experience; the focus should be on educating them to understand the negative life events they face from different angles, actively re-evaluate, think differently, or rationalize emotional incidents.

Second, education regarding gratitude and subjective wellbeing of college students should be strengthened; effective intervention methods such as writing a “gratefulness diary” should be adopted to enhance the awareness of life, and learn how to be grateful. Social comparison theory states that happiness originates from the comparison between reality and standards. When reality is higher than the standards, more pleasure can be obtained. Therefore, in life, students should be guided to be efficient at exploring the positive aspects of things and to treat life with a grateful attitude.

## Limitations and Future Directions

This study has certain limitations: first, the study revealed the mediating role of gratitude and subjective wellbeing on the influence of emotion regulation strategies on the sense of meaning of life through a cross-sectional study; however, it could not make inferences regarding the causal relationship between the variables. It reflected a continuous process of changes in the psychological characteristics of college students’ emotional adjustment, gratitude, subjective wellbeing, and the sense of life meaning. The breadth and depth of the research are limited to a certain extent. In the future, a combination of transverse and longitudinal design could be used to examine the characteristics and changes in-depth. Second, the research adopted the questionnaire survey method, which is relatively simple. Future studies may use experimental research methods to further verify the influence and mechanism of emotion regulation strategies on the sense of life. The results obtained would be more convincing. Third, the research participants were limited to college students in China. Previous study showed the sense of life meaning to be affected by age, culture, and other factors. In the future, studies could be extended to different age ranges and different cultural backgrounds which would be more conducive to the promotion of research results.

Although there are some shortcomings, the research results verified our hypothesis, explained to a certain extent the mechanism of emotion regulation strategies affecting the sense of meaning of life, enriched the previous research results, and provided strong support for educational practitioners to carry out educational activities.

## Data Availability Statement

The raw data to this study will be made available by the authors upon resonable request.

## Ethics Statement

Ethical review and approval was not required for the study on human participants in accordance with the local legislation and institutional requirements. The participants provided their written informed consent to participate in this study.

## Author Contributions

The author confirms being the sole contributor of this work and has approved it for publication.

## Conflict of Interest

The author declares that the research was conducted in the absence of any commercial or financial relationships that could be construed as a potential conflict of interest.

## Publisher’s Note

All claims expressed in this article are solely those of the authors and do not necessarily represent those of their affiliated organizations, or those of the publisher, the editors and the reviewers. Any product that may be evaluated in this article, or claim that may be made by its manufacturer, is not guaranteed or endorsed by the publisher.

## References

[B1] Adler M. G. Fagley N. S. (2005). Appreciation: individual differences in finding value and meaning as a unique predictor of subjective well-being. *J. Pers.* 73 79–114. 10.1111/j.1467-6494.2004.00305.x 15660674

[B2] Balzarotti S. Biassoni F. Villani D. Prunas A. Velotti P. (2016). Individual differences in cognitive emotion regulation: implications for subjective and psychological well-being. *J. Happiness Stud.* 17 125–143. 10.1007/s10902-014-9587-3

[B3] Boden M. T. Bonn-Miller M. O. Kashdan T. B. Alvarez J. Gross J. J. (2012). The interactive effects of emotional clarity and cognitive reappraisal in posttraumatic stress disorder. *J. Anxiety Disord.* 26 233–238. 10.1016/j.jandis.2011.11.007 22169054

[B4] Bono G. Froh J. J. (2009). “Gratitude in school: benefits to students and schools,” in *Handbook of Positive Psychology In Schools*, eds Gilman R. Huebner E. S. Furlong M. J. (New York, NY: Routledge), 77–88.

[B5] Brenning K. Soenens B. Van Petegem S. Vansteenkiste M. (2015). Perceived maternal autonomy support and early adolescent emotion regulation: a longitudinal study. *Soc. Dev.* 24 561–578. 10.1111/sode.12107

[B6] Campos J. J. Campos R. G. Barrett K. C. (1989). Emergent themes in the study of emotional development and emotion regulation. *Dev. Psychol.* 25 394–402. 10.1037/0012-1649.25.3.394

[B7] Chai X. Y. Guo H. Y. Lin D. H. Liu Y. Su S. (2018). The emotion regulation strategies and the psychological well-being among migrant children in china: the roles of self-esteem and resilience. *J. Psychol. Sci.* 41 71–76.

[B8] Chan D. W. (2013). Subjective well-being of hong kong chinese teachers: the contribution of gratitude, forgiveness, and the orientations to happiness. *Teach. Teach. Educ.* 32 22–30. 10.1016/j.tate.2012.12.005

[B9] Chen L. Yuan J. J. He Y. Y. (2009). Emotion regulation strategies: cognitive reappraisal is more effective than expressive suppression. *Adv. Psychol. Sci.* 17 730–735.

[B10] Chu S. T. W. Fung H. H. Chu L. (2019). Is positive affect related to meaning in life differently in younger and older adults? A time sampling study. *J. Gerontol.* 75 2086–2094. 10.1093/geronb/gbz086 31251360

[B11] Crumbaugh J. C. (1973). *Everything to Gain: A Guide to Self-Fulfillment Through Logoanalysis.* Wokingham: Nelson-Hall, 58–60.

[B12] Cutuli D. (2014). Cognitive reappraisal and expressive suppression strategies role in the emotion regulation: an overview on their modulatory effects and neural correlates. *Front. Syst. Neurosci.* 8:175. 10.3389/fnsys.2014.00175 25285072 PMC4168764

[B13] Diener E. (1984). Subject well- being. *Psychol. Bull.* 95 42–75. 6399758

[B14] Disabato D. J. Kashdan T. B. Short J. L. Jarden A. (2017). What predicts positive life events that influence the course of depression? A longitudinal examination of gratitude and meaning in life. *Cogn. Therapy Res.* 41 444–458. 10.1007/s10608-016-9785-x

[B15] Dryman M. T. Heimberg R. G. (2018). Emotion regulation in social anxiety and depression: a systematic review of expressive suppression and cognitive reappraisal. *Clin. Psychol. Rev.* 65 17–42. 10.1016/J.CPR.2018.07.004 30064053

[B16] Duan J. H. (1996). General well-being scale for Chinese college students in the trial results and analysis. *Chinese J. Clin. Psychol.* 4 56–57.

[B17] Fan Z. Y. Wu Y. (2020). Relationship between parent-child relationship, loneliness and depression among the left-behind rural children: gratitude as a mediator and a moderator. *Psychol. Dev. Educ.* 36 734–742.

[B18] Fazio A. F. (1977). *A Concurrent Validation Study of the NCHS General Well-Being Schedule (Dept. of H.E.W. Publ. No HRA-78-1347).* Hyattsville, MD: National Center for Health Statistics.

[B19] Fredrickson B. L. (2001). The role of positive emotions in positive psychology-The broaden-and-build theory of positive emotions. *Am. Psychol.* 56 218–226. 10.1037//0003-066x.56.3.218 11315248 PMC3122271

[B20] Fredrickson B. L. Mancuso R. A. Branigan C. Tugade M. (2000). The undoing effect of positive emotions. *Motiv. Emot.* 24 237–258. 10.1023/a:1010796329158 21731120 PMC3128334

[B21] Fu X. L. Zhang K. (2021). *The Blue Book of Mental Health: Report on the Development of Chinese National Mental Health (2019-2020).* Beijing: Social Sciences Academic Press.

[B22] Garnefski N. Kraaij V. (2011). Relationships between cognitive emotion regulation strategies and depressive symptoms: a comparative study of five specific samples. *Pers. Individ. Diff.* 40 1659–1669.

[B23] Garnefski N. Teerds J. Kraaij V. Legerstee J. Van den Kommer T. (2004). Cognitive emotion regulation strategies and depressive symptoms: differences between males and females. *Pers. Individ. Diff.* 36 267–276. 10.1016/S0191-8869(03)00083-7

[B24] Gong L. Wang X. Q. Qi X. D. (2013). Emotion regulation and life satisfaction: on mediating role of interpersonal disturbances. *J. Southwest China Norm. Univ. (Nat. Sci. Edn.)* 38 145–149.

[B25] Gross J. J. (1998a). The emerging field of emotion regulation: an integrative review. *Rev. Gen. Psychol.* 2 271–299. 10.1037/1089-2680.2.3.271

[B26] Gross J. J. (1998b). Antecedent- and response-focused emotion regulation: divergent consequences for experience, expression, and physiology. *J. Pers. Soc. Psychol.* 74 224–237. 9457784 10.1037//0022-3514.74.1.224

[B27] Gross J. J. (1999). Emotion regulation: past, present, future. *Cogn. Emot.* 13 551–573. 10.1080/026999399379186

[B28] Gross J. J. (2001). Emotion regulation in adulthood: timing is everything. *Curr. Dir. Psychol. Sci.* 10 214–219. 10.1111/1467-8721.00152

[B29] Gross J. J. (2002). Emotion regulation: affective, cognitive, and social consequences. *Psychophysiology* 39 281–291. 10.1017/S0048577201393198 12212647

[B30] Gross J. J. John O. P. (2003). Individual differences in two emotion regulation processes: Implications for affect, relationships, and well- being. *J. Pers. Soc. Psychol.* 85 348–362. 10.1037/0022-3514.85.2.348 12916575

[B31] Guan B. Jepsen D. M. (2020). Burnout from emotion regulation at work: the moderating role of gratitude. *Pers. Individ. Diff.* 56 1–11. 10.1016/j.paid.2019.109703

[B32] Haga S. M. Kraft P. Corby E. K. (2009). Emotion regulation: antecedents and well-being outcomes of cognitive reappraisal and expressive suppression in cross-cultural sample. *J. Happiness Stud.* 10 271–291. 10.1007/s10902-007-9080-3

[B33] Hayes A. F. (2013). *Introduction to Mediation, Moderation, and Conditional Process Analysis. A Regression-Based Approach.* New York, NY: Guilford Press.

[B34] Hayes A. F. (2015). An index and test of linear moderated mediation. *Multivariate Behav. Res.* 50 1–22. 10.1080/00273171.2014.962683 26609740

[B35] Heilman R. M. Crisan L. G. Houser D. Miclea M. Miu A. C. (2010). Emotion regulation and decision making under risk and uncertainty. *Emotion* 10 257–265. 10.1037/a0018489 20364902

[B36] Hicks J. A. Trent J. Davis W. E. King L. A. (2012). Positive affect, meaning in life, and future time perspective: an application of socioemotional selectivity theory. *Psychol. Aging* 27 181–189. 10.1037/a0023965 21707177

[B37] Huang M. E. Guo D. J. (2002). Divergent consequences of antecedent-and response -focused emotion regulation. *Acta Psychol. Sin.* 34 371–380. 10.1037//0022-3514.74.1.224 9457784

[B38] Hughes E. K. Gullone E. Watson S. D. (2011). Emotional functioning in children and adolescents with elevated depressive symptoms. *J. Psychopathol. Behav. Assessment* 33 335–345. 10.1007/s10862-011-9220-2

[B39] Jin Y. C. He M. C. Li J. Y. (2016). The relationship between meaning in life and subjective well-being in China: a meta-analysis. *Adv. Psychol. Sci.* 24 1854–1863.

[B40] Julian F. T. Richard D. L. (2000). A model of neurovisceral integration in emotion regulation and dysregulation. *J. Affect. Disord.* 61 201–216. 10.1016/S0165-0327(00)00338-4 11163422

[B41] King L. A. Hicks J. A. (2006). Positive affect and the experience of sense of life meaning. *J. Pers. Soc. Psychol.* 90 179–196. 10.1037/0022-3514.90.1.179 16448317

[B42] Kleiman E. M. Adams L. M. Kashdan T. B. Riskind J. H. (2013a). Grateful individuals are not suicidal: buffering risks associated with hopelessness and depressive symptoms. *Pers. Individ. Diff.* 55 595–599. 10.1016/j.paid.2013.05.002

[B43] Kleiman E. M. Adams L. M. Kashdan T. B. Riskind J. H. (2013b). Gratitude and grit indirectly reduce risk of suicidal ideations by enhancing meaning in life: evidence for a mediated moderation model. *J. Res. Pers.* 47 539–546. 10.1016/j.jrp.2013.04.007

[B44] Kobylińska D. Zajenkowski M. Lewczuk K. Jankowski K. S. Marchlewska M. (2020). The mediational role of emotion regulation in the relationship between personality and subjective well-being. *Curr. Psychol.* 1–14. 10.1007/s12144-020-00861-7

[B45] Kumar A. Epley N. (2018). Undervaluing gratitude: expressers misunderstand the consequences of showing appreciation. *Psychol. Sci.* 29 1423–1435. 10.1177/0956797618772506 29949445

[B46] Li J. B. Dou K. Liang Y. (2021). The relationship between presence of meaning, search for meaning, and subjective well-being: a three-level meta-analysis based on the sense of life meaning questionnaire. *J. Happiness Stud.* 22 467–489. 10.1007/s10902-020-00230-y

[B47] Li X. (2016). The effects of gratitude on college students’ negative life events and sense of the meaning of life: the moderated mediating effect. *Chinese J. Special Educ.* 3 58–63. 10.3969/j.issn.1007-3728.2016.03.009

[B48] Li Y. He W. Zhang X. Guo F. Cai J. Guo Q. H. (2014). The relationship between parenting style,coping style,index of well-being and life of meaning of undergraduates. *China J. Health Psychol.* 11 1683–1685. 10.1186/s12913-016-1423-5 27409075 PMC4943498

[B49] Lin C. C. (2021). Gratitude and suicidal ideation in undergraduates in taiwan: the mediating role of self-esteem and meaning in life. *Omega J. Death Dying* 84 177–193. 10.1177/0030222819882845 31623525

[B50] Liu S. M. Liu K. T. Li T. T. Lu L. (2015). The impact of mindfulness on subjective well-being of college students: the mediating effects of emotion regulation and resilience. *J. Psychol. Sci.* 38 889–895.

[B51] Liu S. S. Gan Y. Q. (2010). Reliability and validity of the Chinese version of meaning in life questionnaire have revised and formed the Chinese version of the meaning of life scale. *J. Chinese Ment. Health* 24 478–482. 10.1007/s11655-018-2991-5 30484019

[B52] Liu X. Zhao J. X. Shen J. L. (2013). Perceived discrimination and subjective well-being among urban migrant children: the effect of mediator and moderator. *Acta Psychol. Sin.* 45 568–584. 10.3724/SP.J.1041.2013.00568

[B53] Lyubomirsky S. Sheldon K. M. Schkade D. (2005). Pursuing happiness: the architecture of sustainable change. *Rev. Gen. Psychol.* 9 111–131. 10.1037/1089-2680.9.2.111

[B54] Masters J. C. (1991). “Strategies and mechanism for the personal and social control of emotion,” in *The Development of Emotion Regulation and Dysregulation*, eds Garber J. Dodge K. A. (Cambridge: Cambridge University Press), 182–207.

[B55] McCullough M. E. Emmons R. A. Tsang J. A. (2002). The grateful disposition: a conceptual and empirical topography. *J. Pers. Soc. Psychol.* 82 112–127. 10.1037/0022-3514.82.1.112 11811629

[B56] Meng Z. L. (2005). *Emotional Psychology.* Beijing: Peking University Press, 204–205.

[B57] Moore S. Zoellner L. Mollenholt N. (2008). Are expressive suppression and cognitive re-appraisal associated with stress-related symptoms? *Behav. Res. Therapy* 46 993–1000. 10.1016/j.brat.2008.05.001 18687419 PMC2629793

[B58] Ochsner K. N. Bunge S. A. Gross J. J. Gabrieli J. D. E. (2002). Rethinking feelings: an FMRI study of the cognitive regulation of emotion. *J. Cogn. Neurosci.* 14 1215–1229. 10.1162/089892902760807212 12495527

[B59] Peng Y. S. Fang P. Jiang Y. (2011). The status and prospect in research on brain mechanisms of emotion regulation. *J. Psychol. Sci.* 34 1325–1331.

[B60] Quoidbach J. Mikolajczak M. Gross J. J. (2015). Positive interventions: an emotion regulation perspective. *Psychol. Bull.* 141:655. 10.1037/a0038648 25621978

[B61] Rosenberg E. L. (1998). Levels of analysis and the organization of affect. *Rev. Gen. Psychol.* 2 247–270. 10.1037/1089-2680.2.3.247

[B62] Ryff C. D. Singer B. H. (2008). Know thyself and become what you are: a eudaimonic approach to psychological well-being. *J. Happiness Stud.* 9 13–39. 10.1007/s10902-006-9019-0

[B63] Sheppes G. Scheibe S. Suri G. Gross J. J. (2011). Emotion-regulation choice. *Psychol. Sci.* 22 1391–1396. 10.1177/0956797611418350 21960251

[B64] Shrira A. Palgi Y. Ben-Ezra M. Shmotkin D. (2011). How subjective well-being and meaning in life interact in the hostile world? *J. Positive Psychol.* 6 273–285. 10.1080/17439760.2011.577090

[B65] Srivastava S. Tamir M. McGonigal K. M. John O. P. Gross J. J. (2009). The social cost of emotional suppression: a prospective study of the transition to college. *J. Pers. Soc. Psychol.* 96 883–897. 10.1037/a0014755 19309209 PMC4141473

[B66] Steger M. F. Frazier P. Oishi S. Kaler M. (2006). The meaning in life questionnaire: assessing the presence of and search for meaning in life. *J. Couns. Psychol.* 53 80–93. 10.1037/0022-0167.53.1.80

[B67] Steger M. F. Oishi S. Kashdan T. B. (2009). Meaning in life across the life span: levels and correlates of meaning in life from emerging adulthood to older adulthood. *J. Positive Psychol.* 4 43–52. 10.1080/17439760802303127

[B68] Strumpfer D. J. W. (2006). Positive emotions, positive emotionality and their contribution to fortigenic living: a review. *Home South Afr. J. Psychol.* 36 144–167.

[B69] Szczygie D. Mikolajczak M. (2017). Why are people high in emotional intelligence happier? They make the most of their positive emotions. *Pers. Individ. Diff.* 117 177–181. 10.1016/j.paid.2017.05.051

[B70] Tamirb M. (2016). Why do people regulate theire motions? A taxonomy of motives in emotion regulation. *Pers. Soc. Psychol. Rev.* 20 199–222. 10.1177/1088868315586325 26015392

[B71] Thompson R. A. (1994). Emotion regulation: a theme in search of definition. The development of emotion regulation: biological and behavioral considerations. *Monogr. Soc. Res. Child Dev.* 59 25–52. 10.1111/j.1540-5834.1994.tb01276.x 7984164

[B72] Tongeren D. R. V. Green J. D. Davis D. E. Hook J. N. Hulsey T. L. (2015). Prosociality enhances meaning in life. *J. Positive Psychol.* 11 1–12. 10.1080/17439760.2015.1048814

[B73] Wang L. Liu H. C. Li Z. Q. Du W. (2017). Reliability and validity of emotion regulation questionnaire Chinese revised version. *China J. Health Psychol.* 15 503–505.

[B74] Wang Z. H. Guo D. J. (2003). Review of gross’s research on emotion regulation process and strategy. *Adv. Psychol. Sci.* 11 629–634.

[B75] Watkins P. C. Woodward K. Stone T. Kolts R. L. (2003). Gratitude and happiness: development of a measure of gratitude and relationships with subjective well-being. *Soc. Behav. Pers.* 31 431–452. 10.2224/sbp.2003.31.5.431

[B76] Wei C. Wu H. T. Kong X. N. Wang H. T. (2011). Revision of gratitude questionnaire- 6 in Chinese adolescent and its validity and rellability. *Chinese J. Sch. Health* 10 1201–1202.

[B77] Witvliet C. Richie F. J. Luna L. M. R. Van Tongeren D. R. (2018). Gratitude predicts hope and happiness: a two study assessment of traits and states. *J. Posit. Psychol.* 14 271–282. 10.1080/17439760.2018.144924

[B78] Wood A. M. Joseph S. Linley P. A. (2007). Coping style as a psychological resource of grateful people. *J. Soc. Clin. Psychol.* 26 1108–1125. 10.1016/j.cjtee.2020.11.007 33308964 PMC7878450

[B79] Wood A. M. Joseph S. Maltby J. (2008). Gratitude uniquely predicts satisfaction with life: incremental validity above the domains and facets of the five factor model. *Pers. Individ. Diff.* 45 49–54. 10.1016/j.paid.2008.02.019

[B80] Xie X. L. Zou B. (2013). Mediating effect of subjective well-being on attitude towards commit suicide and meaning in life in poverty-stricken university students. *Shanghai Jiaotong Univ. (Med. Sci.)* 33 78–83.

[B81] Xu C. Xu Y. Xu S. Zhang Q. Liu X. Shao Y. (2020). Cognitive reappraisal and the association between perceived stress and anxiety symptoms in COVID-19 isolated people. *Front. Psychiatry* 11:858. 10.3389/fpsyt.2020.00858 32982809 PMC7492600

[B82] Yin Z. M. Sun M. Wang Y. (2019). Optimism brings meaning of life to college students: partial mediation effect of well-being. *Adv. Psychol.* 5 904–910.

[B83] Zhu R. R. Gan Y. Q. Li Y. Zhang X. (2017). Mediating effect of positive emotion on relationship between cognition reappraisal and meaning of life in freshmen. *J. Chinese Ment. Health* 31 490–494.

